# Global Conformational Dynamics of HIV-1 Reverse Transcriptase Bound to Non-Nucleoside Inhibitors 

**DOI:** 10.3390/biology1020222

**Published:** 2012-07-26

**Authors:** David W. Wright, Benjamin A. Hall, Paul Kellam, Peter V. Coveney

**Affiliations:** 1 Centre for Computational Science, Department of Chemistry, University College London, London, WC1H 0AJ, UK; Email: dave.wright@ucl.ac.uk (D.W.W.); benjamin.a.hall@ucl.ac.uk (B.A.H.); 2 The Wellcome Trust Sanger Institute, Wellcome Trust Genome Campus, Hinxton, UK; Email: pk5@sanger.ac.uk

**Keywords:** HIV-1, reverse transcriptase, non-nucleoside reverse transcriptase inhibitor (NNRTI), nevirapine, efavirenz, molecular dynamics, elastic network model

## Abstract

HIV-1 Reverse Transcriptase (RT) is a multifunctional enzyme responsible for the transcription of the RNA genome of the HIV virus into DNA suitable for incorporation within the DNA of human host cells. Its crucial role in the viral life cycle has made it one of the major targets for antiretroviral drug therapy. The Non-Nucleoside RT Inhibitor (NNRTI) class of drugs binds allosterically to the enzyme, affecting many aspects of its activity. We use both coarse grained network models and atomistic molecular dynamics to explore the changes in protein dynamics induced by NNRTI binding. We identify changes in the flexibility and conformation of residue Glu396 in the RNaseH primer grip which could provide an explanation for the acceleration in RNaseH cleavage rate observed experimentally in NNRTI bound HIV-1 RT. We further suggest a plausible path for conformational and dynamic changes to be communicated from the vicinity of the NNRTI binding pocket to the RNaseH at the other end of the enzyme.

## 1. Introduction

Since its discovery in the early eighties AIDS, the clinical result of infection by HIV has caused over 25 million deaths worldwide [1]. The global reach and fatal prognosis of human immunodeficiency virus (HIV) infection have emphasised the need for effective antiretroviral therapies. While several effective treatment regimens have been devised, involving inhibitors that target multiple viral proteins [2], the emergence of mutations in these proteins is a contributing factor to the eventual failure of treatment. Understanding the interactions of inhibitory drugs and viral proteins may in the future allow us to tailor treatment to individual patients and develop more effective therapies. 

**Figure 1:**
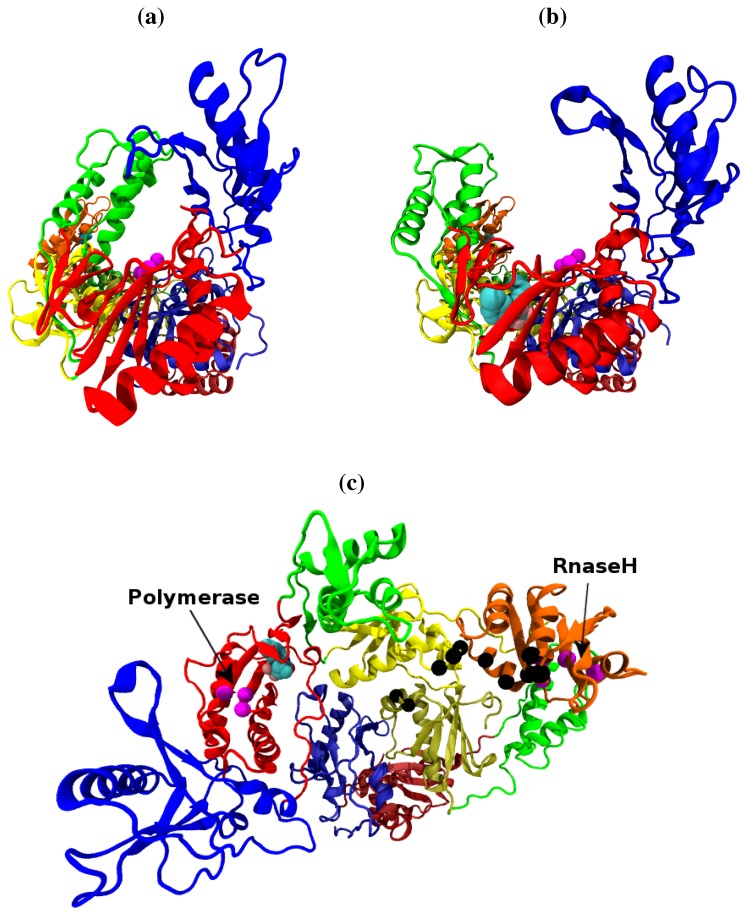
The structure of the HIV-1 RT shown along the template/primer binding cleft in the (**a**) closed apo (based on the 1DLO crystal structure) and (**b**) open NNRTI bound conformation (based on the EFV bound 1IKW structure, the van der Waals surface of the drug is shown). In this view the p66 subunit is most prominent, in (**c**) viewed from above the template/primer binding cleft both the p66 and p51 monomers are clearly visible. The subunits are named after the structures supposed likeness to a right hand. It is in fact, the folding of the p66 seen in (a) and (b) which results in the likeness. However the subdomains retain their name in p51 as seen in (b). The subdomains are known as the (F)ingers (blue), (P)alm (red), (T)humb (green), (C)onnection (yellow) and (R)NaseH (orange). The first 4 subdomains form the polymerase domain and are common to both subunits. In all figures the residues of the polymerase and RNaseH active sites are shown as magenta beads (the former is located in the palm). The residues of the RNaseH primer grip are indicated by black beads.

The essential role played by reverse transcriptase (RT) in the replicative cycle of HIV has made it an attractive target for HIV/AIDS therapy. The enzyme uses the single stranded RNA viral genome as a template to create a single strand of DNA which is in turn used as a template to create a double stranded DNA (*ds*DNA) copy of the genome. The *ds*DNA copy is then suitable for integration into the chromosomes of human host cells. HIV-1 RT is a multifunctional enzyme with distinct polymerase and RNaseH active sites. At the polymerase active site incoming nucleotides matching the template RNA or DNA are incorporated into the growing complementary DNA chain. The RNaseH active site catalyses the breakdown of the RNA genome, freeing the DNA copy to act as a template for the creation of the final double stranded DNA genome. Inhibition of either enzymatic activity compromises viral replication. Two classes of inhibitor are currently in clinical use, called nucleotide analogue RT inhibitors (NRTIs) and non-nucleoside analogue RT inhibitors (NNRTIs). The former mimic the natural deoxynucleotide triphosphate (dNTP) substrate, binding to nascent DNA chains and acting as chain terminators preventing further elongation. NNRTIs operate allosterically, binding approximately 10 Å from the polymerase active site in a pocket which does not exist in the apoenzyme (see Figure 1). 

HIV-1 RT is a heterodimeric protein consisting of 440 and 560 amino acid subunits (known as p51 and p66 respectively due to their molecular weights). Both subunits are derived from the same *gag-pol* polyprotein with the p51 subunit lacking the C-terminal domain (containing the RNaseH active site), which is cleaved from it by the HIV-1 protease. The PDB [3] contains many different crystal structures of HIV-1 RT in a range of states including those bound to both NRTIs [4,5] and NNRTIs [6,7,8], natural DNA and RNA substrates [9,10,11,12] in addition to the apoenzyme [13]. The supposed resemblance of the p66 to a right hand has led to the shared subdomains being known as the fingers, palm, thumb and connection [14]. The reported crystal structures of the unliganded enzyme show the tips of the p66 fingers and thumb domains in close proximity, with a separation of less than 15 Å (one structure does not exhibit this feature but was created by soaking out an NNRTI), in what is known as the closed conformation. Despite deriving from the same sequence the subunits have very different forms once folded. All template/primer bound systems show the enzyme in a more open configuration with the template/primer substrate fitting between the thumb and fingers domains [15]. A similar conformation, but with greater separation between the p66 fingers and thumb, is seen in all NNRTI bound structures. Figure 1 shows both the closed apo and open NNRTI bound conformations of the enzyme. Both the polymerase and RNase H catalytic sites are located within the p66 subunit. The polymerase active site (formed by Asp110, Asp185 and Asp186) is found in the palm subdomain, the RNaseH active site being located at the opposite end of the enzyme with a distance of approximately 30 Å separating them along the central template/primer binding cleft. When bound to a RNA/DNA hybrid template/primer approximately 19 base pairs are contained between the two catalytic sites [12]. The template/primer is positioned at the two active sites by contacts with regions known as the polymerase primer grip (p66 residues 227 to 235) and the RNaseH primer grip (p66 residues 359, 360, 361, 473, 475, 476, 501 and 505, and p51 residues 395 and 396) [12]. 

Several mechanisms by which NNRTIs may inhibit reverse transcriptase have been proposed. Two of the most widely described are that NNRTI binding disrupts the geometry of the polymerase active site [16], or that the conformation or dynamics of the polymerase primer grip are perturbed preventing catalytically competent positioning of the 3^'^-end of the primer. Another common model is the “arthritic thumb” model in which NNRTI binding is said to restrict the movement of the p66 thumb [17,18,19]. The reduction of thumb motility is thought to prevent the translocation of the template/primer complex during nucleic acid polymerisation. Recently, the need to reconcile data indicating that NNRTIs can influence the rate of RNaseH processing [20,21] and the clinical observation of resistance mutations distal from the NNRTI binding pocket [22,23,24] have led to the realisation that a more subtle picture accounting for bidirectional coupling between the two active sites is necessary for a full understanding of NNRTI function. 

**Figure 2:**
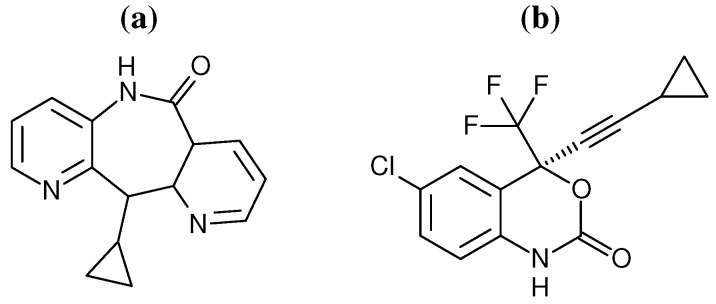
Chemical structures of the two NNRTIs used in this study: (**a**) nevirapine (NVP) and (**b**) efavirenz (EFV).

In contrast to the wealth of structural data comparatively little experimental evidence is available describing the conformation and dynamics of the entire enzyme in solution that could help to reach this understanding. Spin labelling experiments have shown that in solution the enzyme exists in an equilibrium between this state and a more open conformation [25], and more recently hydrogen exchange mass spectrometry (HXMS) has been used to provide information on the dynamics of the peptide backbone [26,27]. Similarly the relatively large size of HIV-1 RT has limited the number and scope of computational studies. Recently, studies have begun to emerge that probe NNRTI binding [28,29,30] and its impact on enzyme dynamics at the atomistic level. In particular molecular dynamics (MD) simulations by Ivetac and McCammon [29] have provided evidence to support the “arthritic thumb” hypothesis. However, coarse grained network model analysis, based on crystal structures, has indicated that different NNRTIs alter domain movements in distinctive ways. Here we use a combination of network models and atomistic MD to investigate the impact of the binding of two different NNRTIs on the dynamics of distant regions of the HIV-1 RT structure. The inhibitors efavirenz (EFZ) and nevirapine (NVP) were chosen to represent the NNRTI class of drugs (the chemical structures of both drugs are shown in Figure 2). 

## 2. Methods

### 2.1. Anisotropic Network Model

We applied the anisotropic network model (ANM) as described in detail in Eyal *et al.* [31] and implemented in the ProDy package [32] to all available HIV-1 RT crystal structures bound to NVP and EFV. Briefly, all C_α_ atoms from a structure are regarded as nodes within a network and are regarded as being bound to one another by springs if they are within a given spatial cut off distance. A uniform force constant γ is adopted for all springs. A Hessian matrix is generated by considering the individual Cartesian components of each spring, resulting in a 3N × 3N matrix (where N is the number of residues). The resulting matrix is solved for eigenvalues and eigenvectors which provide information regarding the mean square fluctuations of individual residues and correlations between them (6 zero eigenvalue modes are generated representing translation and rotation). We followed Temiz and Bahar [33] and use a cut-off of15 Å and γ =1. 

Correlation between modes generated from different structures was calculated from the dot product of eigenvectors representing each mode (considering only common residues and ensuring that the eigenvectors were normalised before the calculation was performed), producing values between 1 (correlated) and −1 (anti-correlated). No meaning is attached to the direction of a mode, so correlated or anti-correlated modes are both equivalent. 

### 2.2. Principal Component Analysis

Principal component analysis is a dimensional reduction technique that allows the isolation of the most significant conformational differences between a set of structures. Here the structures are provided by snapshots from the molecular dynamics trajectory. The correlation matrix is calculated from an aligned molecular dynamics trajectory and then diagonalised. This provides an orthogonal set of eigenvectors representing linearly independent modes of conformational change called principal components. The eigenvalues associated with each principal component are a measure of the variance in the original dataset described by that component. The principal component analysis presented here was performed on the C_α_ coordinates of a range of crystal structures using the ProDy package [32] in order to identify the conformational differences which distinguish them. 

### 2.3. Ensemble Molecular Dynamics

Three HIV-1 RT structures were selected to be simulated using molecular dynamics. These were the 1DLO apo structure, the 1IKW EFV bound structure and 1VRT NVP bound structure. For each structure an ensemble of 10 independent simulations were performed (differing only in the initial velocities applied to each atom), each producing 4 ns of production simulation (each simulation lasted a total of 10 ns including equilibration). Structures were output for analysis every 10 ps. 

#### 2.3.1. Model Creation

Unfortunately, all available HIV-1 RT crystal structures are incomplete and a number of loop residues in the p51 subunit of the NNRTI bound structures are missing (residues 217 to 231 in 1IKW, 88 to 92 and 215 to 231 in 1VRT, and 356 to 362 in both structures). The models were completed by copying in the coordinates from 1HQU [6] (this structure was chosen due to its high resolution (2.7 Å) and the fact it was bound to an NNRTI) after alignment of the surrounding residues using VMD [34]. In each case the final model contains 556 residues in the first (p66) chain and 427 in the second (p51) chain for a total of 983 residues (the sequence of all simulated structures was the same). Once the manual editing of structures was complete the rest of the simulation workflow was automated using the Binding Affinity Calculator (BAC) scripts created to automate simulations and free energy calculations for the HIV-1 protease [35]. Each system was solvated using a cubic box of TIP3P water molecules [36] with at least 14 Å distance around the protein. The systems were neutralised by the addition of Cl^− ^ions. 

Inhibitor potential parameterisation was performed by extracting the drug coordinates into separate files, using the PRODRG tool [37] to insert missing hydrogens. The geometries were then optimised using Gaussian 98 [38] (with the 6-31G** basis functions). The Restrained Electrostatic Potential (RESP) procedure, part of the AMBER package [39], was used to calculate the partial charges. The force field parameters for the inhibitors were described using the General AMBER Force Field (GAFF) [40]. The protein and DNA elements of all systems were described by the standard AMBER force field (ff03) [41] which is parameterised for bio-organic molecules and including DNA in particular. The default variants (such as protonation states) for amino acids in physiological conditions were used for all residues. 

#### 2.3.2. Simulation Protocol

The molecular dynamics package NAMD2 [42] was used throughout the minimisation, equilibration and production stages of the simulations. Electrostatic interactions were treated using the particle mesh Ewald (PME) [43] method and SHAKE [44] constraints were applied to all bonds involving hydrogen atoms in order to employ a 2 fs integration time step. Minimisation was conducted using the conjugate gradient and line search algorithms for 2,000 iterations of each system. During this process all heavy atoms were restrained using a force constant of 5 kcal mol^−^^1^Å^2 ^. 

The next stage of the equilibration process was a mutational relaxation protocol in which each mutated residue and residues within 5 Å are released in turn from the restraints for 50 ps. This allowed the residues to reorient into more favourable conformations if necessary. After the 50 ps relaxation period the restraints are reapplied to each region. 

The equilibration phase anneals the system taking the temperature from 50 K to 300 K in 50 ps. Once achieved, the final temperature was maintained using a Langevin thermostat with a coupling coefficient of 5 ps^−^^1 ^. This was followed by completely isothermal equilibration for 200 ps in the canonical (NVT) ensemble. In both of these stages the restraints imposed during minimisation were retained. The restraints were then gradually reduced in four steps of 1 kcal mol^−^^1^Å^2^, each step running for 50 ps. The restraints applied are weaker than in the protease case as no regions are known to suffer solvation induced deformities unlike the flap region of the protease. After this, the restraints were removed completely and the systems allowed to evolve under isothermal-isobaric (NPT) conditions using a Berendsen barostat [45] with a target pressure of 1 bar and a pressure coupling constant of 0.1 ps. Coordinate trajectories were recorded every 1 ps throughout all equilibration and production runs. 

The three systems were built using extensions to the Binding Affinity Calculator (BAC) scripts created to automate simulations and free energy calculations for the HIV-1 protease [35]. The Application Hosting Environment (AHE) [46] was used in order to automate the running of simulations and retrieval of data. 

#### 2.3.3. Equilibration

Physical properties can only be reliably calculated from systems which have been adjudged properly equilibrated. For all systems simulated here the potential energy minimisation applied is sufficient to remove all bad contacts as measured by the decrease in potential energy which, after the heating phase, remains stable with a standard deviation of less than 450 kcal mol^−^^1 ^in all cases. A further test of whether the systems under study have equilibrated was made by investigating the structural variation seen over the simulation. Using difference distance matrices, Keller *et al.* [47] determined a set of residues which vary in relative position by less than 2 Å in a wide range of HIV-1 RT crystal structures. These residues are assumed to represent the most structurally stable regions of the protein (listed in Supporting Information Supplementary Table S2). The root mean squared fluctuations (RMSF) of each system was calculated relative to the average structure for these residues (HIV-1 RT is known to be a flexible protein with a number of loop regions for which conformational changes might be expected throughout even equilibrated simulations). The RMSF of the simulations reduced to around 1.5 Å after 6 ns for all systems. Consequently the trajectory between the start of the simulation and 6 ns in is defined as being the equilibration phase and all subsequent parts of the simulation comprise the so-called production phase. All analyses presented here are derived from production phase simulations. 

## 3. Results: Coarse Grained Models of HIV-1 RT Dynamics

**Figure 3:**
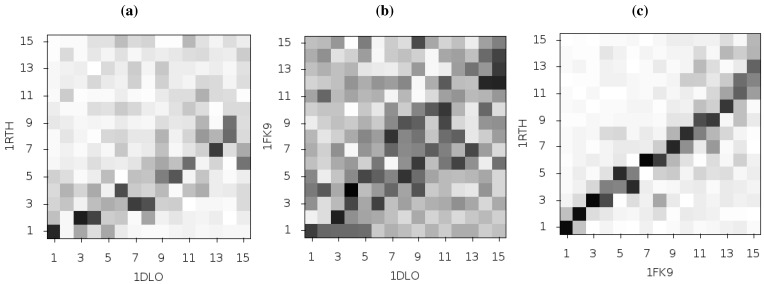
Comparison of the first 15 ANM modes generated from the apo HIV-1 RT structure and those of the NVP and EFV bound enzyme using the 1DLO, 1RTH and 1FK9 crystal structures respectively. The matrices show the pairwise correlation of modes, the darker the square the higher the magnitude of the correlation. (**a**) The apo to NVP bound and (**b**) apo to EFV bound comparisons show similar patterns with high correlations between the first and third modes and lower correlation in most other modes. (**c**) shows that the modes for the EFV and NVP bound structures are much more similar. The correlation of mode 2 for both structures with other modes indicates that this mode is not identical between the two structures.

**Figure 4:**
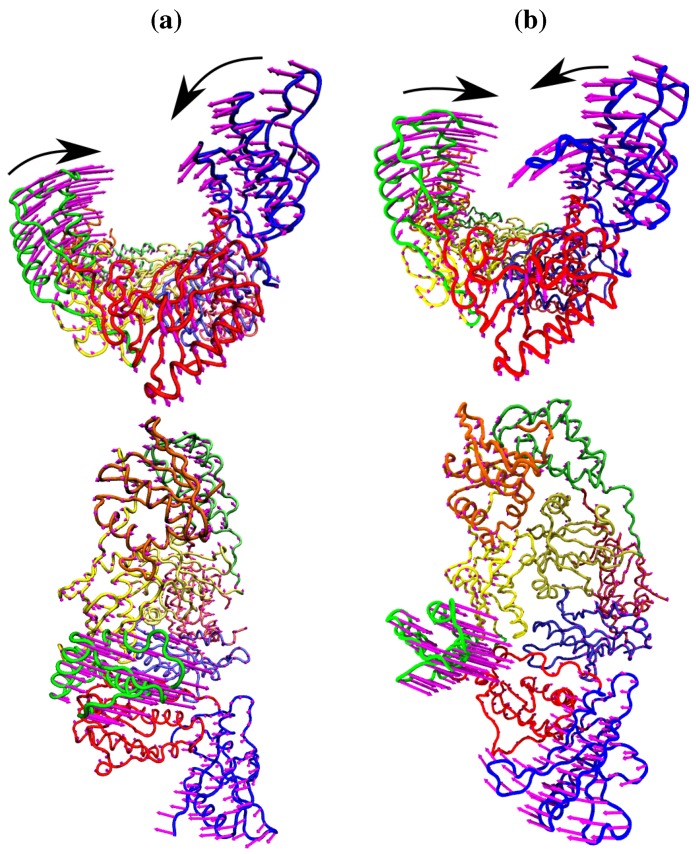
The fluctuations of the NNRTI bound HIV-1 RT described by the second ANM mode for (**a**) the EFV bound 1FK9 and (**b**) NVP bound 1RTH structures. The fluctuations of each residue are shown as magenta arrows. In both cases two views are shown, one above the other; along the DNA binding cleft and looking down into it. In both cases the motions are dominated by anti-correlated movements of the p66 thumb and fingers (as highlighted by the black arrows).

**Figure 5:**
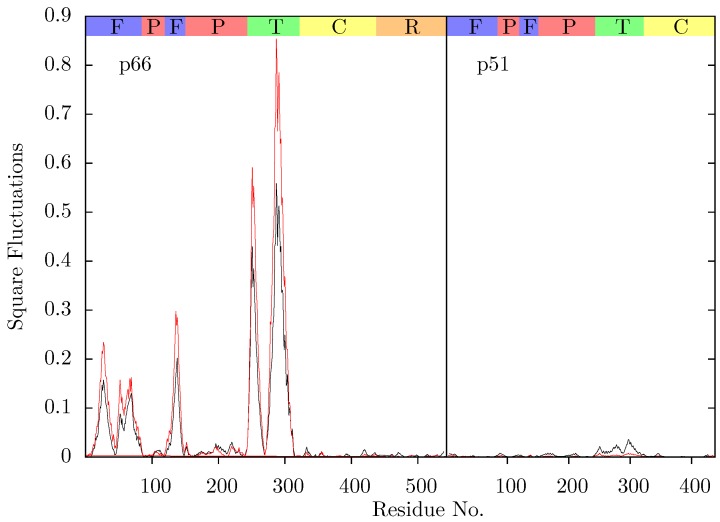
Comparison of the squared fluctuations predicted by ANM mode 2 of the NVP bound 1RTH (red) and EFV bound 1FK9 structures (black).

Temiz and Bahar [33] suggest that differences in the second ANM mode calculated for NPV and EFV bound HIV-1 RT may be linked to the differing efficacy of the two drugs. This was based on analysis of the 1RTH NVP bound and 1FK9 EFV bound PDB crystal structures. The modes for both structures were observed to differ noticeably from those computed for the apo 1DLO structure. Figure 3 shows the pairwise correlation between the first 15 modes calculated for all three of these structures. In each comparison darker squares indicate higher magnitude of correlation between two individual modes and hence that the shape of the motions represented is similar (there is no physical meaning to the direction of the mode and consequently no meaning attached to a whether a pair of modes are correlated or anti-correlated). A dark diagonal in a heat map is indicative that not only are the modes generated for both structures similar but that their rank ordering is also the same. Clear differences between the two NNRTI bound structures and the apo structure are seen (in Figure 2a,b), with only a clear correlation between mode 1 of both classes of structure and between mode 3 of the apo and mode 2 of the drug bound structures apparent. In contrast, there are strong correlations close to the diagonal in Figure 2c indicating many of the modes generated for 1RTH and 1FK9 are very similar, and are similarly ordered. The second mode describes anti-correlated motions of the fingers and thumb in both cases (see Figure 4), suggestive of the possibility that the NNRTI bound HIV-1 RT can adopt a closed conformation despite this not having been observed crystallographically. Despite the qualitative similarities the comparison of this mode between the two systems exhibits some level of correlation with a range of other modes, reflecting the differences observed by Temiz and Bahar [33]. There are three principle sources of these differences which are illustrated in Figure 5; these are reduced motions of the p66 fingers (residues 1 to 84 and 120 to 150) and thumb (residues 244 to 322), along with additional motions in the p51 domain (between residues 170 and 260) of the EFV bound structure relative to that bound to NVP. 

### 3.1. Influence of Initial Crystal Structure Choice

**Figure 6:**
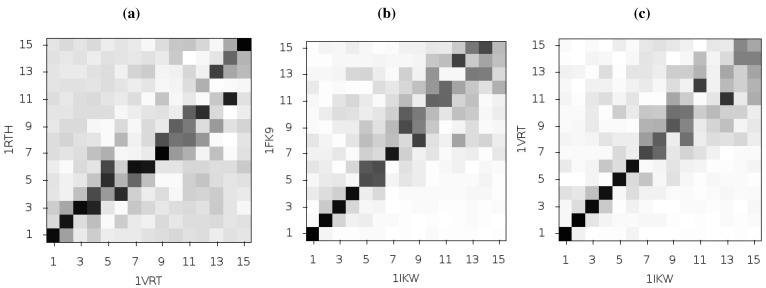
Comparison of the first 15 ANM modes generated from different HIV-1 RT structures bound to NVP and EFV, using the 1RTH and 1VRT NVP bound structures and 1FK9 and 1IKW EFV bound structures. The matrices show the magnitude of the pairwise correlation of the modes, the darker the square the higher the correlation. (**a**) and (**b**) show comparisons of the modes generated from different crystal structures but with RT bound to the same inhibitor, NVP and EFV respectively. In (**c**) the 1IKW (EFV) and 1VRT (NVP) bound structures are compared, more of the low modes show high levels of similarity than in the plot for 1FK9 and 1RTH shown in Figure 2c.

**Figure 7:**
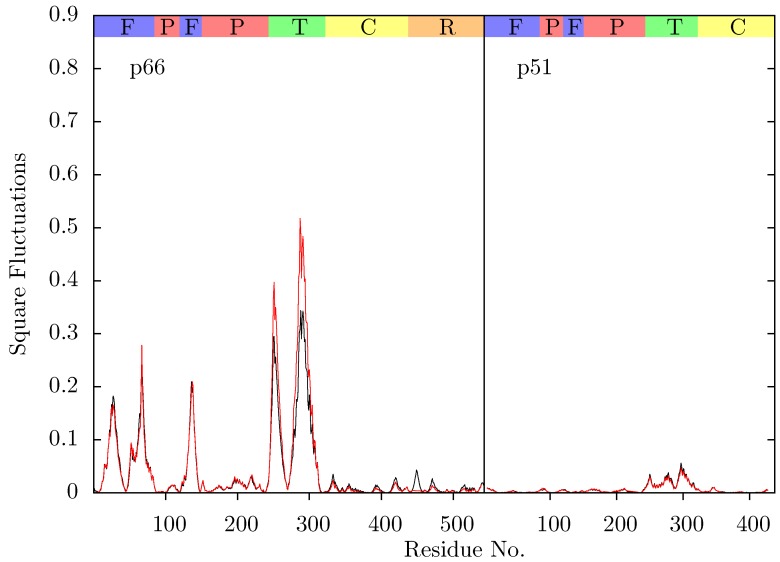
Comparison of the squared fluctuations predicted by ANM mode 2 of the NVP bound 1VRT (red) and EFV bound 1IKW structures (black). The main difference between the two structures is in the extent of the predicted mobility of the p66 thumb (residues 244 to 322); in both cases this is much than that observed for 1RTH and 1FK9 (see Figure 5).

If the differences observed between the modes seen for 1FK9 and 1RTH is caused by differences in the bound inhibitor, then similar differences should be observed using different EFV and NVP bound structures. We used all available EFV and NVP crystal structures as inputs to ANMs. We found that whilst all structures showed similar second modes, there was no correlation between the particular drug bound and the predictions of the flexibility of the p66 thumb and fingers, or the presence or absence of fluctuations in the p51 subunit. A comparison between the results for the 1IKW and 1VRT crystal structures (EFV and NVP bound, respectively) and those of 1FK9 and 1RTH (EFV and NVP bound, respectively) is shown in Figure 6. The NVP bound 1VRT and 1RTH structures in particular show dissimilarities across a range of modes. Comparison of the fluctuations described by mode 2 for 1IKW and 1VRT shown in Figure 7 indicates that not only are the motions more restrained in these models but both exhibit fluctuations in p51, removing the clear distinction seen between 1FK9 and 1RTH. Furthermore, the inclusion or exclusion of a node representing the drug, placed at the drug centre of mass as in [33], is not found to impact results (see Supporting Information Supplementary Figure S2). 

**Figure 8:**
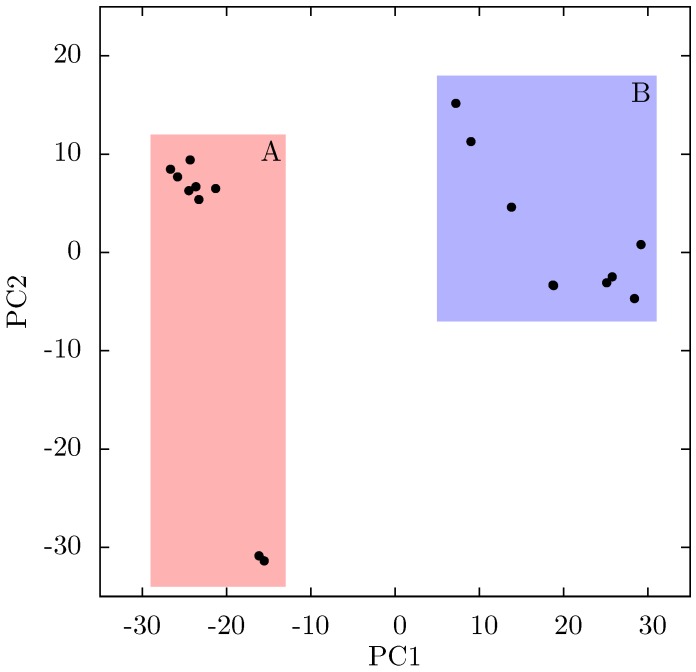
Projection of individual crystal structures on to the first two principal components generated from PCA of EFV and NVP bound HIV-1 RT structures. The structures are clearly clustered into two groups along PC1, labelled A and B, and highlighted in red and blue respectively.

If there is no link between the specific inhibitor bound and the differences seen in the ANM modes of NNRTI bound HIV-1 RT, the obvious question is which features of the structures are causing the observed variation. In order to assess these differences observed in the ANMs we used PCA to analyse the differences in the available EFV and NVP bound structures. Analysis of all available crystal structures revealed one prominent principal component (PC), but this only identified differences between the 3HVT structure which was crystalised in the *C*2 space group and the other crystal structures all in the *P* 2_1_2_1_2_1_ space group. This difference is known to produce alterations in RT conformation [48] and the fact this structure is also anomalous in not having a water molecule bound beside NVP led us to exclude it from consideration here. The first PC generated for the reduced dataset accounts for 48% of the variance observed and the second a further 20% (the variance accounted for by the first 20 PCs is shown in Supporting Information Supplementary Figure S4). The projection of each crystal structure analysed onto these two components is shown in Figure 8. Two separate clusters of structures are apparent in this data, where we have labelled the two groups A and B. All structures in group A (including 1FK9,1IKW and 1VRT) show p51 flexibility in their second ANM modes, whereas those in group B do not. A list of structures in each group is provided in Table 1. Both groups contain both EFV and NVP structures and both wild type and resistant sequences (although NVP bound structures with the key NNRTIBP mutations Y181C and Y188C are all found in group B). The two structures separated from the main group A along PC2 cluster (Figure 8) are 1IKV and 1IKW. The variation along PC2 is dominated by motions in highly flexible loops in the p66 fingers (see Supporting Information Supplementary Figure S5). As these two structures were produced in the same study [7] it is likely that these minor changes are a function of the particular crystallization protocol employed. 

**Figure 9:**
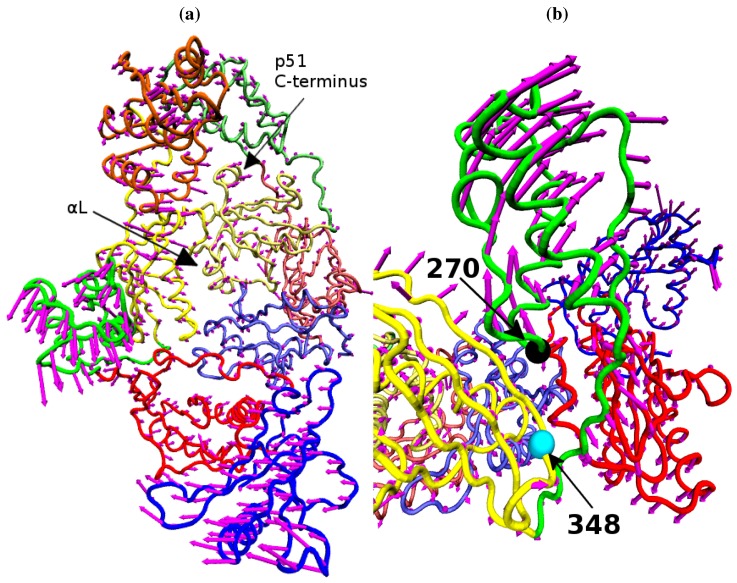
The variation of the NNRTI bound HIV-1 RT described by PC1. The variation of each residue, relative to the average structure displayed in tube representation, is shown in (**a**) as magenta arrows. Detail of the changes in their p66 thumb is shown in (**b**), the C_α_ atoms of residue 270 (black) and 348 (cyan) are shown as spheres.

Figure 9 shows that structural variation along PC1 is dominated by changes in the position of the p66 thumb and fingers, albeit much more subtle changes than seen between the open and closed conformations of HIV-1 RT. The most notable change is the rotation of the helix *αH* in the thumb, which changes approximately 15° across the data set (see Figure 8b); this is accompanied by the movement of the loop running from residue 269 to 278 (between *αI* and *αJ*). A range of smaller alterations along the template/primer binding cleft of the protein is also observed. Three regions in p51 show significant changes compared to surrounding residues; the C-terminus (residues 420 to 430), *αJ* in the p51 thumb and *αL*. Furthermore, p51 *αL* contains residues Lys395 and Glu396 of the RNaseH primer grip. The regions of high variation seen in PC1 suggest a path for the communication of conformational changes around the p66 thumb (and NNRTI binding pocket at its base) to distal regions of the protein. This path would see changes in the thumb communicated directly to the connection domain via the nearest residues, 348 and 355, through the loop between p66 *β*18 (which contains residue 355) and on to the dimer interface between the RNaseH and the p51 C-terminal residues and thumb. Interestingly, residue 348 which provides contacts between the thumb and connection domains is one of a number of connection domain residues for which resistance associated mutations have been identified [22,23]. 

**Table 1:** The EFV and NVP bound HIV-1 RT crystal structures analysed by PCA are divided into two groups according to PC1, groups A and B. Group A corresponds to the structures in which p51 flexibility is linked to that of the p66 thumb by ANMs, no such link is observed in ANMs based on group B structures. The drug bound and major resistance associated mutations present in the structure are also listed.

PDB code	NNRTI	Resolution (Å)	Resistance Mutations	Citation
**Group A: p51 flexible **				
LW0	NVP	2.80	T215Y	Chamberlain *et al.* [49]
LWC	NVP	2.62	M184V	Chamberlain *et al.* [49]
S1U	NVP	3.00	L100I	Ren *et al.* [50]
VRT	NVP	2.20	–	Ren *et al.* [51]
HND	NVP	2.50	K101E	Ren *et al.* [52]
HNY	NVP	2.50	E138K	Ren *et al.* [52]
FK9	EFV	2.50	–	Ren *et al.* [53]
IKV	EFV	2.00	K103N	Lindberg *et al.* [7]
IKW	EFV	3.00	–	Lindberg *et al.* [7]
**Group B: p51 constrained **				
FKP	NVP	2.90	K103N	Ren *et al.* [53]
JLB	NVP	3.00	Y181C	Ren *et al.* [54]
JLF	NVP	2.60	Y188C	Ren *et al.* [54]
LWE	NVP	2.81	M41L, T215Y	Chamberlain *et al.* [49]
LWF	NVP	2.80	M41L, D67N, K70R, M184V, T215F, K219N	Chamberlain *et al.* [49]
RTH	NVP	2.20	–	Ren *et al.* [51]
S1X	NVP	2.80	V108I	Ren *et al.* [50]
FKO	EFV	2.90	K103N	Ren *et al.* [53]
JKH	EFV	2.50	Y181C	Ren *et al.* [54]

## 4. Results: Ensemble Molecular Dynamics

**Figure 10:**
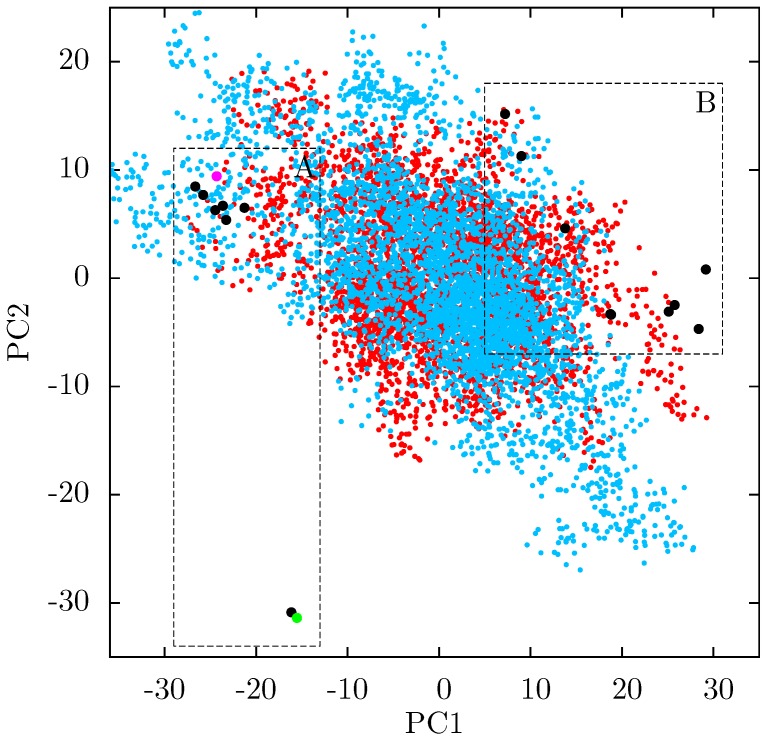
Projection of snapshots from the ensemble MD trajectories of the EFV and NVP bound HIV-1 RT onto the first two principal components generated from PCA of EFV and NVP bound HIV-1 RT crystal structures. Both simulations show wide variations in both PCs; there is no clustering close to either of the groups of crystal structure projections (these are shown by the dashed rectangles and labelled A and B). Crystal structure projections are shown as black dots, except the 1IKW (green) and 1VRT (magenta) structures which were used as initial structures for the EFV and NVP MD simulations, respectively.

Both the PCA and ANM analyses presented here suggest a link between alterations in p66 thumb position and changes in distal regions of the protein implicated in RNaseH function. This combination of evidence is suggestive of NNRTI binding inducing changes in both conformation and dynamics throughout the protein. Whilst predictions from ANMs do give consistently similar global motions for the proteins, we have seen that small changes in local contacts can influence the details of the dynamic modes produced. In order to gain a more detailed picture of the dynamics in the p51 subunit, and how it is influenced by NNRTI binding, we conducted ensemble MD simulations of the apo HIV-1 RT and the same enzyme bound to both NVP and EFV. Figure 10 shows that both the NVP and EFV bound simulations move away from their initial structures and sample conformations which differ from both the group A and B structures seen in the crystal structures. This is indicative of the fact that the p66 thumb and fingers in particular are constrained by crystal contacts in the experimental structures of NNRTI bound HIV-1 RT. 

**Figure 11:**
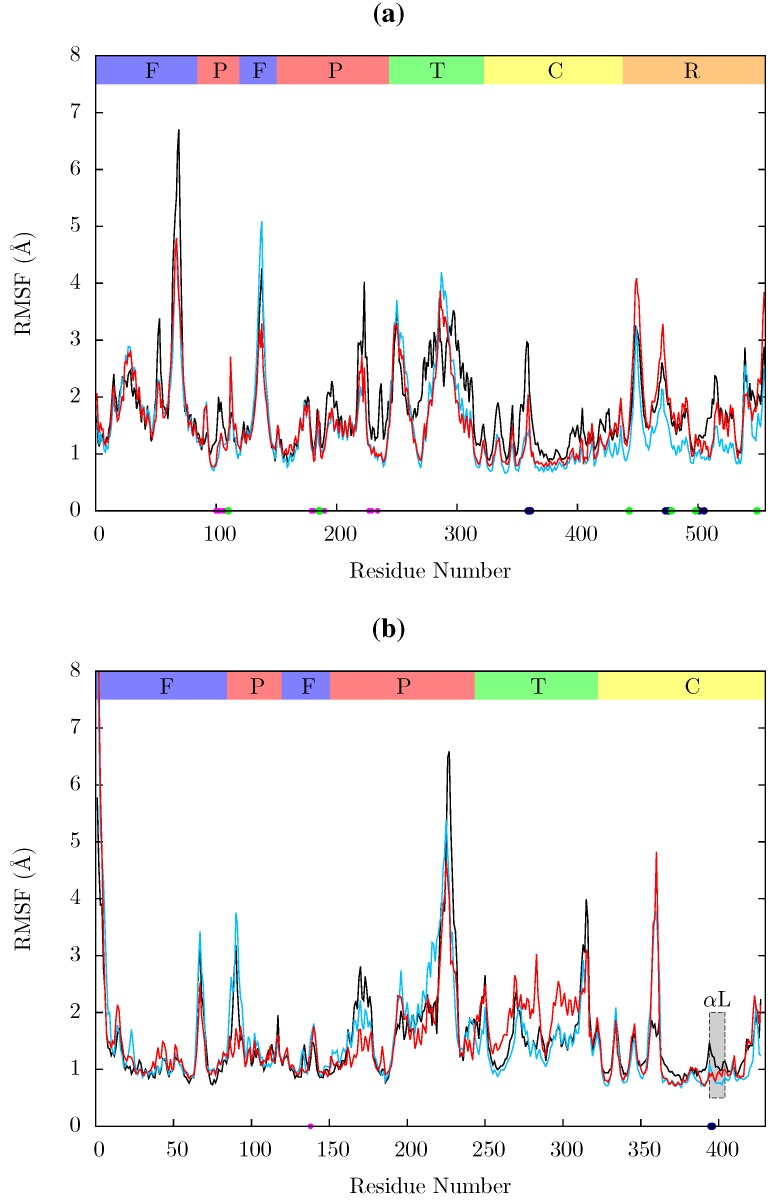
Root mean squared fluctuations (RMSF) of the C_α_ atoms of (a) p66 and (b) p51 over the concatenated ensemble trajectories of the apo (black), EFV (red) bound and NVP (blue) bound HIV-1 RT simulations. The residues of the NNRTI binding pocket, RnaseH primer grip and active sites are indicated by magenta, dark blue and green dots on the x-axis, respectively. The bars above the graph indicate the locations of the subunits in the sequence; the (F)ingers, (P)alm, (T)humb, (C) connection and (R)NaseH. The p51 αL helix containing residues 395 and 396 in the RNaseH primer grip is indicated by a gray box.

**Figure 12:**
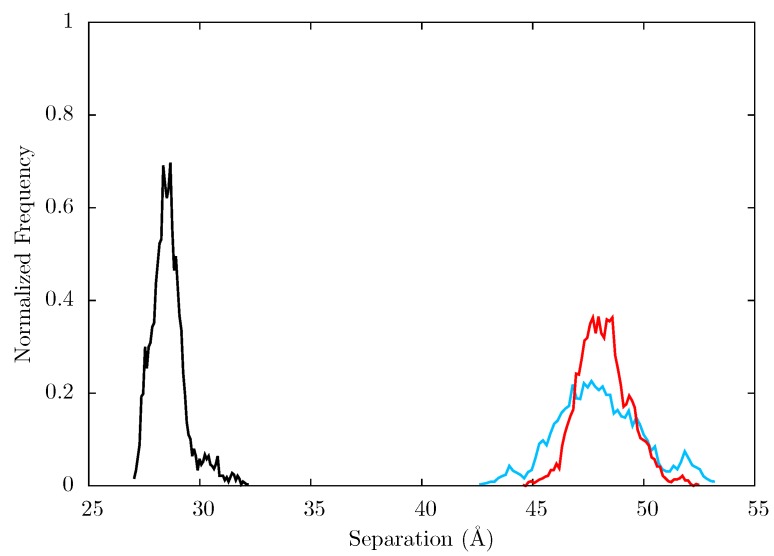
Histogram showing the normalized frequency distribution of the separation between centres of mass of the p66 fingers and thumb over the simulations of the (black), EFV (red) bound and NVP (blue) bound HIV-1 RT ensemble trajectories. Both the EFV and NVP ensembles maintain much larger separation than is seen in the apo enzyme but the NVP bound system shows greater flexibility.

Figure 11 shows the root mean squared fluctuations (RMSF) of the residues of the three simulated structures relative to the average structure of each set of trajectories. As expected from both ANM models and crystal structure evidence [55], the regions in which the largest motions are observed for all simulated structures are the p66 fingers and thumb subdomains. However, no shifts between open and closed global conformations occur in any simulations such as those reported by Ivetac and McCammon [29] (see Figure 12) as being observed in 4 member 30 ns ensemble simulations (we are describing 10 member 10 ns ensembles here). The residues that constitute the NNRTI binding pocket as expected all exhibit markedly lower flexibility in the two drug bound simulations. Residues 320 to 405 in p66 are also stabilized in the NVP and EFV bound simulations, in line with evidence from hydrogen/deuterium exchange experiments performed by Seckler *et al.* [27] which suggests that EFV binding suppresses motions of the connection domain. Distinct differences are seen in the RNaseH domain between the two NNRTI bound ensembles. The NVP bound enzyme exhibits reduced flexibility throughout the domain compared to the apo system. A variety of changes are observed in the EFV bound system with residues between 443 (which forms part of the RNaseH primer grip) and 481 increasing in flexibility; most of the rest of the domain exhibits similar behaviour to the apo system with residues 500 to 516 (including residues 501 and 505 in the RNaseH primer grip) being stabilized. 

Residues 86 to 91 (*β*5) in p51 are stabilized in the EFV bound system (again in line with Seckler *et al.* [27]); NVP however exhibits very similar behaviour to the apo system in this region. The p51 thumb in EFV is more flexible than in either the NVP bound or apo structure (this is the region predicted to exhibit fluctuations correlated with thumb and fingers motions in the ANM). The p51 thumb is adjacent to the RNaseH domain and it has been suggested that it plays a role in maintaining its correct conformation [56]. However, we do not observe generally increased RNaseH domain flexibility in our simulations of the EFV bound system but instead more subtle changes. Both NNRTI bound structures show increased flexibility around p51 residue 358 (in a loop connecting *β*18 to *αK*). 

**Figure 13:**
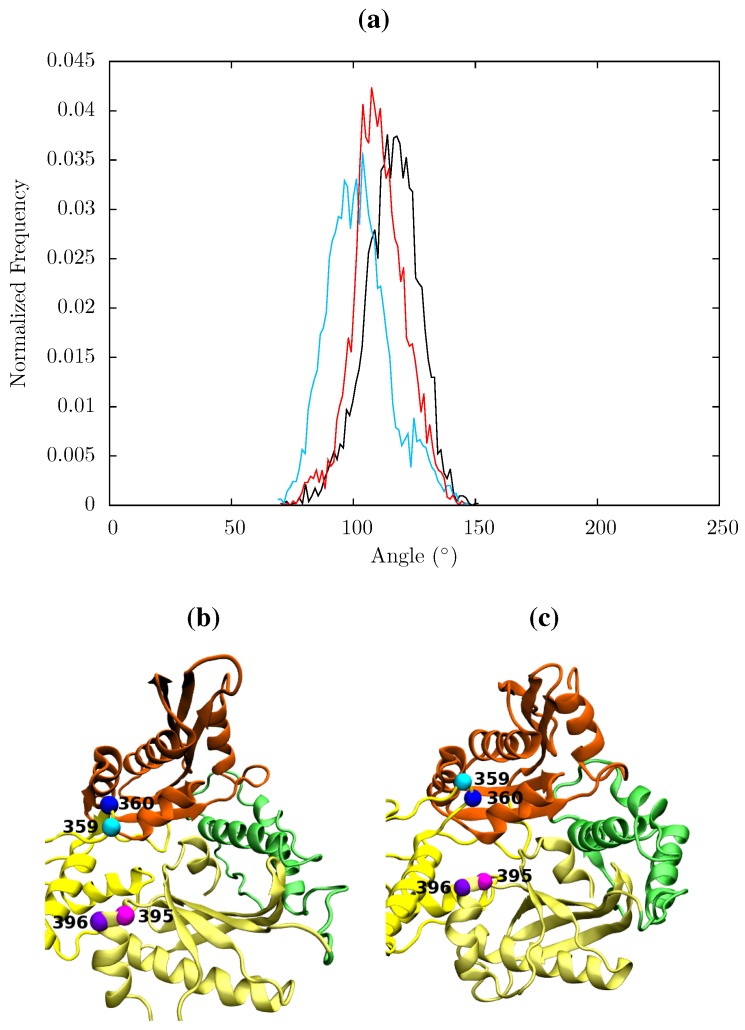
(**a**) Histogram showing the normalized frequency distribution of the angle between two pairs of residues (359 and 360 in p66 and 395 and 396 in p51) in the RNaseH primer grip over the simulations of the apo (black), EFV (red) bound and NVP (blue) bound HIV-1 RT ensemble trajectories. The angle is measured between two vectors joining the C_α_ atoms of the pairs of residues. The distribution is shifted to a lower angle in both NNRTI bound structures. Conformations of the RNaseH primer grip residue pairs 359 and 360 in p66 (shown in cyan and blue, respectively) and 395 and 396 in p51 (in magenta and purple, respectively) are illustrated in (**b**) from the NVP bound simulation (angle 90°) and (**c**) form the apo simulation (angle 130°). This shows the that the variation is mainly due to flexibility in the loop containing residues 359 and 360 in p66.

In all structures the section between residues 360 and 420 in p51 is highly stable but there is a distinct RMSF peak at RNaseH primer grip residues 395 and 396 in the apoprotein. This key region for substrate positioning was also picked out in the PCA of the EFV and NVP bound crystal structures as undergoing conformational shifts correlated to changes in the p66 thumb. Further investigation of the surrounding regions indicates that other regions in or close to the RNaseH primer grip also undergo discernibly different motions in our simulations, in particular the p66 loop containing residue 359 exhibits very different behaviour across the systems (see Figure 11). Measurements of the angle made between the backbone of residues 395 and 396 with that of residues 359 and 360 shows a shift in both NNRTI bound systems. These changes differentially increase the accessibility of the RNaseH active site, with NVP. The differences between the apo and NNRTI bound structures here are mostly because conformational changes occur to residues 359 and 360 with relatively modest changes in residues 395 and 396 (see Figure 13). 

**Figure 14:**
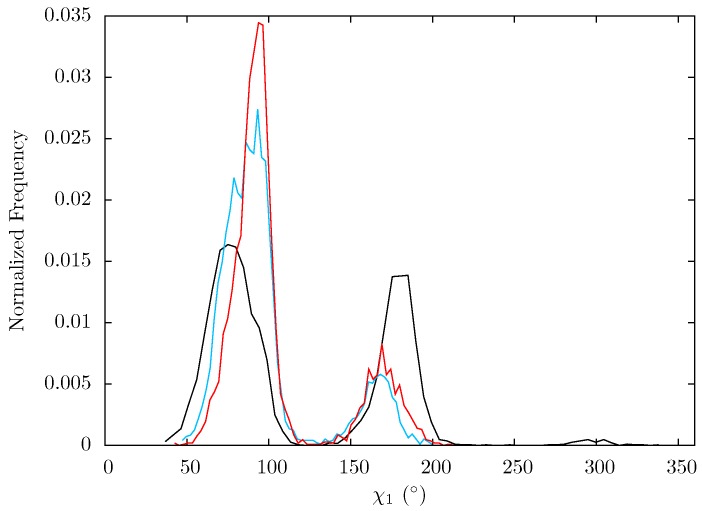
Histogram showing the normalized frequency distribution of the sidechain dihedral angle χ1 of Glu396 over the simulations of the apo (black), EFV (red) bound and NVP (blue) bound HIV-1 RT ensemble trajectories. Two states are apparent for all three structures, however the balance in occupation is shifted to that with a lower angle in the EFV and NVP bound simulations.

In order to ascertain whether the relatively minor changes in backbone flexibility impact residues Lys395 and Glu396, the dihedrals of the sidechains were measured for each snapshot of all three ensembles. The distribution of angles observed for Lys395 are very similar for all three systems (see Supporting Information Supplementary Figure S6) but there is a distinct shift in χ1 for Glu396. Figure 14 shows that in all three systems two states exist. In the apo system the first of these is centred around 75° and the second 180° which are both fairly equally populated. In both NNRTI bound systems the mean angles of these two states are shifted towards one another by approximately 20° and the lower of these two groups is enriched compared to the higher. Changes at this position are particularly significant as in structures of the RNA/DNA bound enzyme [12] Glu396 is seen to interact with the RNA primer backbone phosphate between the eleventh and twelfth base pairs from the 3^' ^end of the DNA primer (positioned at the polymerase active site). This location coincides with the location at which “polymerization-independent” hydrolysis is observed to occur experimentally [20]. The absence of substrate in our simulations means we cannot directly assess the impact of the observed changes on template/primer conformations. However, it seems likely that changes to the binding cleft would be reflected in changes in substrate binding and there is considerable evidence that RNaseH functioning is sensitive to changes in substrate conformation [20,57,58]. Experiments involving the neutralisation of the backbone sections that contact p51 residues 395 and 396 enhanced the rate of cleavage 19 and 21 base pairs from the 3^' ^end of the primer when contact with 396 was lost (no change was seen for 395) [20]. 

## 5. Discussion and Conclusions

Both the anisotopic network models (ANMs) and ensemble molecular dynamics simulations presented here indicate that NNRTI binding induces changes in the dynamics of regions of the protein far from the binding site. ANMs are computationally cheap and have been shown to replicate global dynamics derived from other sources for a number of proteins [55] but our results suggest that caution should be exercised when using them to generate detailed predictions of protein dynamics. In particular we have demonstrated that ANM modes in HIV-1 RT are sensitive to the addition of a small number of contacts between the p66 thumb and connection domain. Specifically we have shown that the predicted motions of regions of the p51 subunit in the second ANM mode of NNRTI bound HIV-1 RT are significantly altered by distal contacts between the thumb and the region close to residue 348 in the p66 connection subdomain. Mutation of residue 348 is implicated in NNRTI resistance pathways; using PCA we have identified a plausible route for dynamic changes to be propagated from residues in the vicinity of the p66 thumb to distant areas of the enzyme that interact with the RNaseH domain. We propose that changes in the p66 thumb could propagate through direct contacts to residues 348 and 355, and on via the loop joining p66 *β*18, through the dimer interface to the RNaseH, the p51 C-terminal residues and thumb. This provides a potential explanation for the effects of mutation Asn348Ile which is observed to restore RNaseH function to normal rates, reversing the acceleration observed in the presence of NVP (the mutation cannot restore correct functioning in the presence of EFV) [59]. 

The atomic detail of our ensemble molecular dynamics simulations allows us to identify subtle shifts in the conformational states of residues in the RNaseH primer grip induced by NNRTI binding. In particular we showed that in both NVP and EFV bound systems the distribution of dihedral angles explored by Glu396 is altered, whilst those of Lys395 remain unaltered despite the shared changes to backbone flexibility. This observation is significant as Glu396 has been shown experimentally to alter RNaseH processivity which is found to be insensitive to the removal of contacts with residue Lys395 [20]. Future studies could look to expand on the findings presented here through the use of extended single trajectories and more complicated free energy calculations (such as PMF or umbrella sampling). We hope that the link demonstrated here between various regions of HIV-1 RT will form part of a move towards general understanding of the allosteric connection between the different domains of the enzyme which may in turn generate insights that pave the way for quantitative analysis of the impact of resistance associated mutations, much as qualitative studies applied to the HIV-1 protease system [60] have led to successful drug binding calculations [61,62,63]. 

## Supplementary Files

Supplementary File 1 Supplementary Materials (PDF, 3241 KB)
