# Supplementary material for: Global Conformational Dynamics of HIV-1 Reverse Transcriptase Bound to Non-Nucleoside Inhibitors
Source: Biology (Basel). 2012 Jul 26;1(2):222–44. doi: 10.3390/biology1020222 (PMC4009785; doi:10.3390/biology1020222)
Supplement: Supplementary File 1 — Supplementary Materials (PDF, 3241 KB) [file biology-01-00222-s001.pdf]

In the main article we describe the investigation of the impact on the global dynamics of HIV-1 RT of the binding of two drugs of the NNRTI class. We use two main techniques, coarse grained ANM models and atomistic MD.

1. HIV-1 RT Secondary Structure

The labelling of the different secondary structure elements of HIV-1 RT is widespread in the literature. In Table S1 we present a table containing the consensus elements and their positions in the amino acid sequence of both the p51 and p66 subunits as originally set out by Jacobo-Molina *et al.* [1].

Table S1. The RT subunits are named after the structures supposed likeness to a right hand. It is in fact the folding of the p66 seen in (**a**) that results in the likeness. However the subdomains retain their name in p51 as seen in (**b**). The secondary structure elements and their position in the amino acid sequence of both subunits are shown in (**c**).

Secondary Structure	p66	p51	Secondary Structure	p66	p51
**Fingers (1–84)**			**Thumb (244–322)**		
β0	7–12	7–12	αH	255–268	254–270
β1	18–24	19–22	αI	278–286	277–283
αA	28–44	28–44	αJ	298–311	289–310
β2	49–51	49–51	β15	316–321	316–321
β3	56–63	56–63			
β4	73–77	72–76	**Connection (323–437)**		
αB	78–83	78–84	β16	326–333	325–333
			β17	336–341	336–343
**Palm (85–119)**			β18	350–358	350–358
β5a	86–90	87–90	αK	364–382	364–381
β5b	94–96	94–96	β19	388–391	386–392
β6	105–112	105–112	αL	395–404	395–404
αC	114–117	112–115	β20	406–412	410–416
			β21	421–424	–
**Fingers (120–150)**			β22	427–430	–
αD	122–127	122–127			
β7	128–134	128–134	**RNaseH (438–560)**		
β8	141–147	141–147	$\beta 1'$	438–447	
			$\beta 2'$	452–459	
**Palm (151–243)**			$\beta 3'$	462–470	
αE	155–174	155–174	$\alpha A'$	474–488	
β9	178–183	179–183	$\beta 4'$	492–497	
β10	186–191	186–191	$\alpha B'$	500–508	
αF	195–212	198–212	$\alpha D'$	516–527	
β11a	214–217	214–219	$\beta 5'$	530–536	
β11b	219–222	–	$\alpha E'$	544-555	
β12	227–229	–			
β13	232–235	–			
β14	238–242	239–242			

2. Equilibration

[2] used difference distance matrices to determine a set of residues which varied in relative position by less than 2 Å in a wide range of HIV-1 RT crystal structures. These residues, assumed to represent the most structurally stable regions of the protein, were used to assess simulation equilibration and are listed in Table S2. Consequently the trajectory between the start of the simulation and 6 ns in is defined as being the equilibration phase and all subsequent parts comprise the production phase.

Table S2. Residues determined to vary relative positions by less than 2 Å in a survey of HIV-1 RT crystal structures by [2].

Chain	Residues
p66	4–6, 95–107, 162–163, 180–181, 188–200, 202–205, 226, 234–235, 237–239, 317, 319, 323, 339–345, 349–353, 365–366, 368–402, 405–419, 428–436, 439, 493, 530
p51	6–7, 18–45, 54–64 71–84, 97–111, 113–117, 121, 123–138, 140–174, 176–184, 186–192, 197–198, 201–202, 208, 252, 254–264, 267, 274, 277, 280–282, 284, 296, 298–300, 303–307, 320, 322, 329, 331, 333–335, 364–393, 397–417

3. Anisotropic Network Models

In the main text images of ANM mode 2 were provided for 1FK9 and 1RTH as this provided the focus of the text. Figure S1 shows the motions described by ANM mode 1; in both cases this is dominated by the correlated motions of the p66 fingers and thumb domains.

Figure S1. The fluctuations of the NNRTI bound HIV-1 RT described by the first ANM mode for (**a**) the EFV bound 1FK9 and (**b**) NVP bound 1RTH structures. The fluctuations of each residue are shown as magenta arrows. In both cases the motions are dominated by correlated movements of the p66 thumb and fingers.

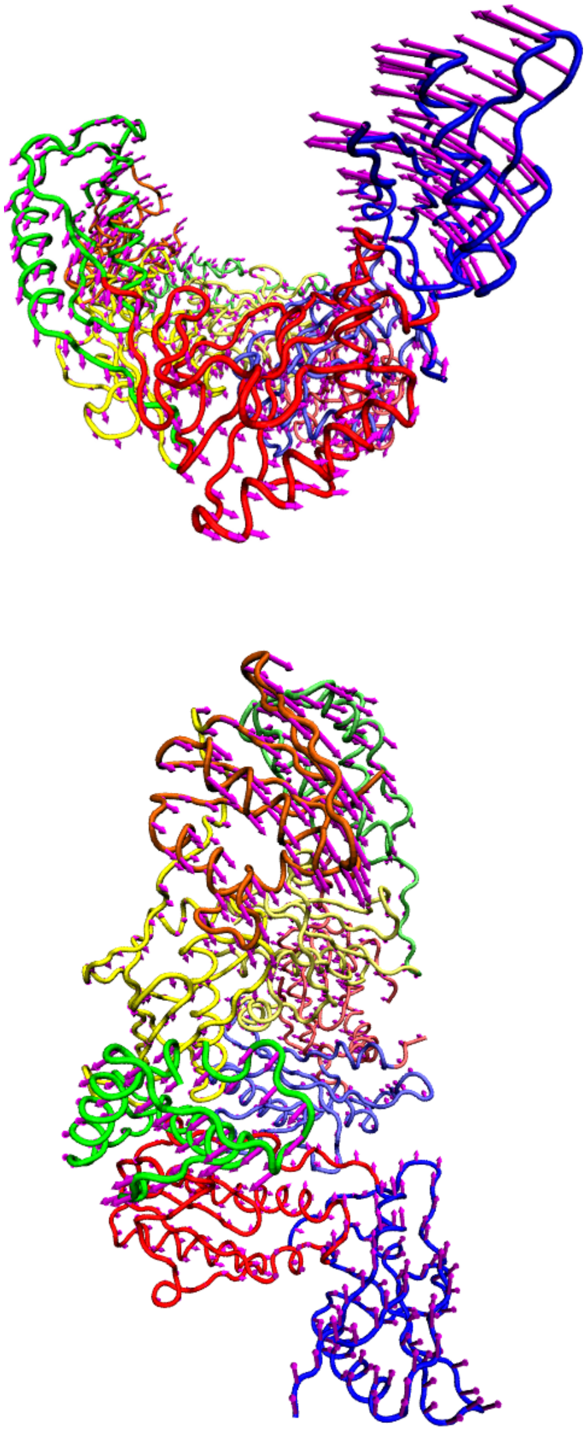

(**a**) (**b**)

The correlation of the first 15 ANM modes for the 4 crystal structures featured in the main text with and without a node included at the centre of mass of the bound NNRTI is shown in Figure S2. In all cases the first 10 modes are identical.

Figure S2. Comparison of the first 15 ANM modes generated from NVP and EFV bound HIV-1 RT structures with and without a node included at the centre of mass of the bound NNRTI. The structures analysed are (**a**) 1RTH; (**b**) 1FK9; (**c**) 1VRT and (**d**) 1IKW. The matrices show the correlation of pairs of modes, the darker the square the higher the correlation. Positive correlations are shaded red, negative black.

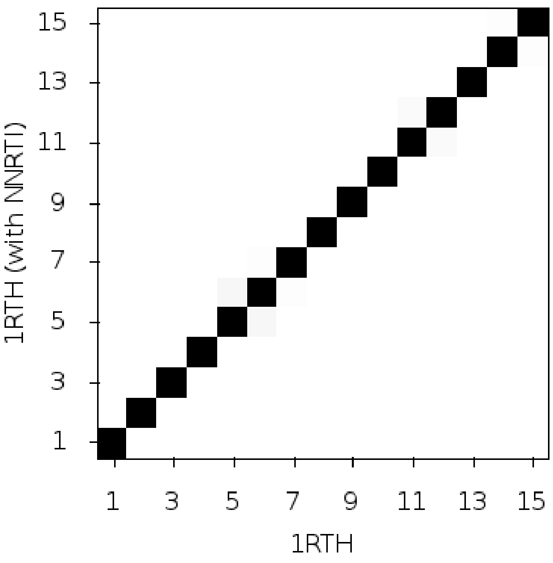

(**a**)

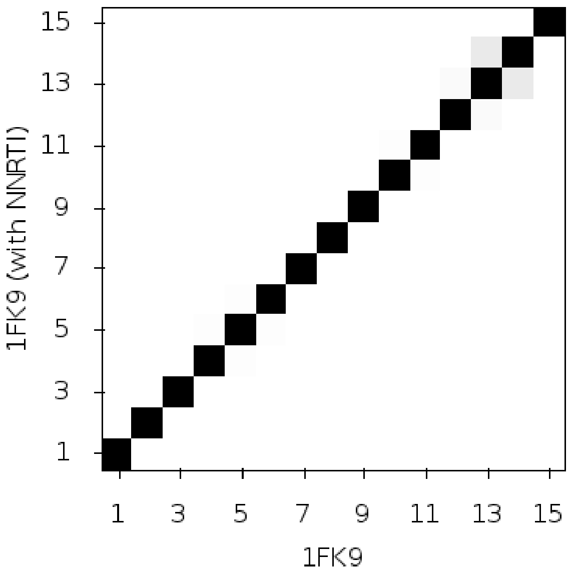

(**b**)

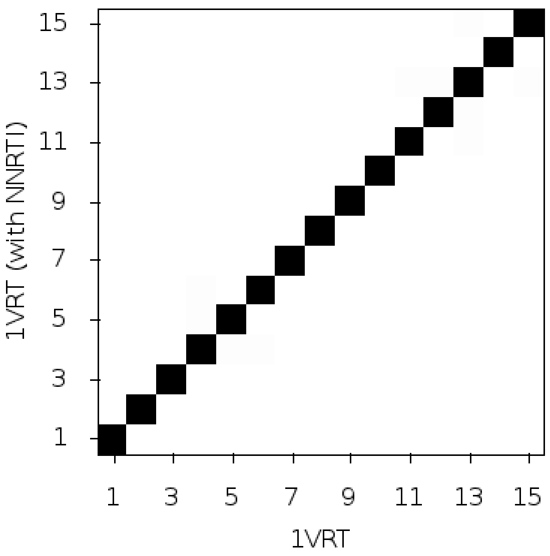

(**c**)

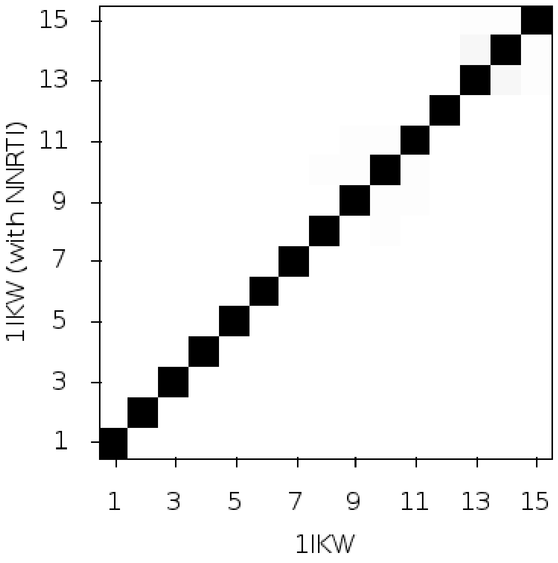

(**d**)

Figure S3 shows the comparison of the ANMs generated for the NNRTI bound crystal structures 1IKW and 1VRT with that of the 1DLO apo structure.

Figure S3. Comparison of the first 15 ANM modes generated from the apo HIV-1 RT structure and those of the NVP and EFV bound enzyme using the 1DLO, 1VRT and 1IKW crystal structures respectively. The matrices show the correlation of pairs of modes, the darker the square the greater the magnitude of the correlation.

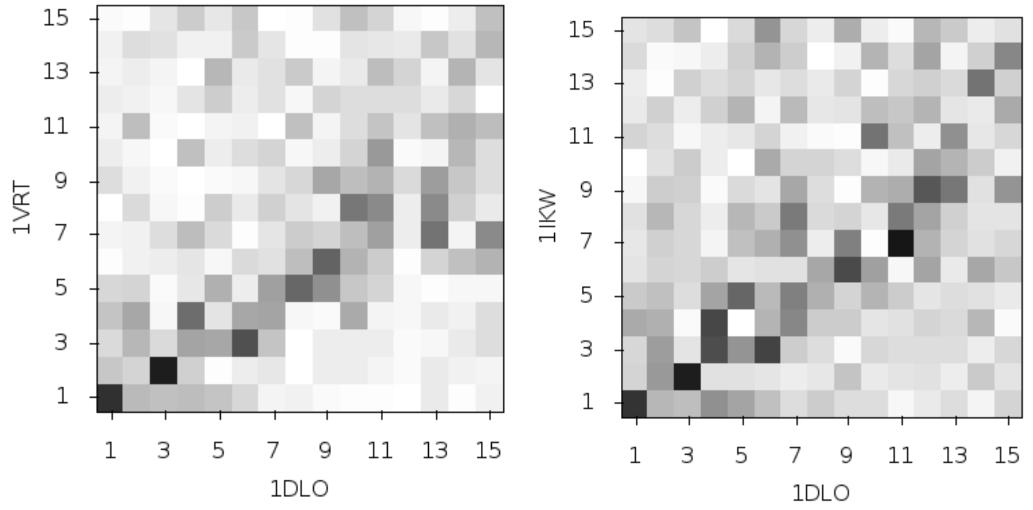

(**a**) (**b**)

4. Principal Component Analysis

The percentage of variance in the set of all EFV and NVP bound crystal structures described by each PC generated, alongside the projections of each crystal structure on PC1 and PC3, is shown in Figure S4. PC3 is the last mode which describes more than 10% of the overall variance. No neat grouping corresponding to that group A and group B ANM models is apparent in the grouping of the PC3 projections. The structural variation represented by each PC is shown in Figure S5.

Figure S4. (**a**) Percentage of variance described by each PC generated from PCA of EFV and NVP bound HIV-1 RT structures; (**b**) Projections of all crystal structures onto PC1 and PC2. The structures are clearly clustered into two groups along PC1.

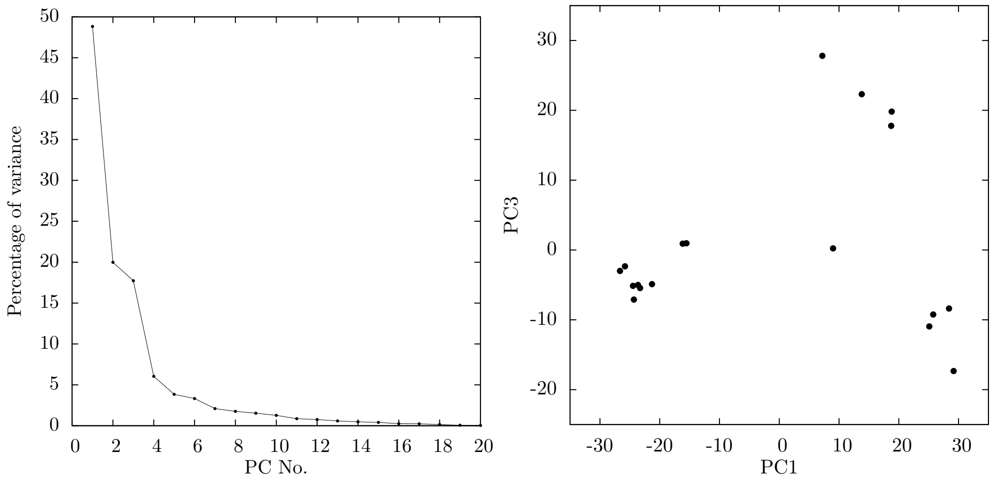

(**a**) (**b**)

Figure S5. The variation of the NNRTI bound HIV-1 RT described by (**a**) PC1; (**b**) PC2 and (**c**) PC3. The variation of each residue, relative to the average structure displayed in tube representation, is shown in (**a**) as magenta arrows.

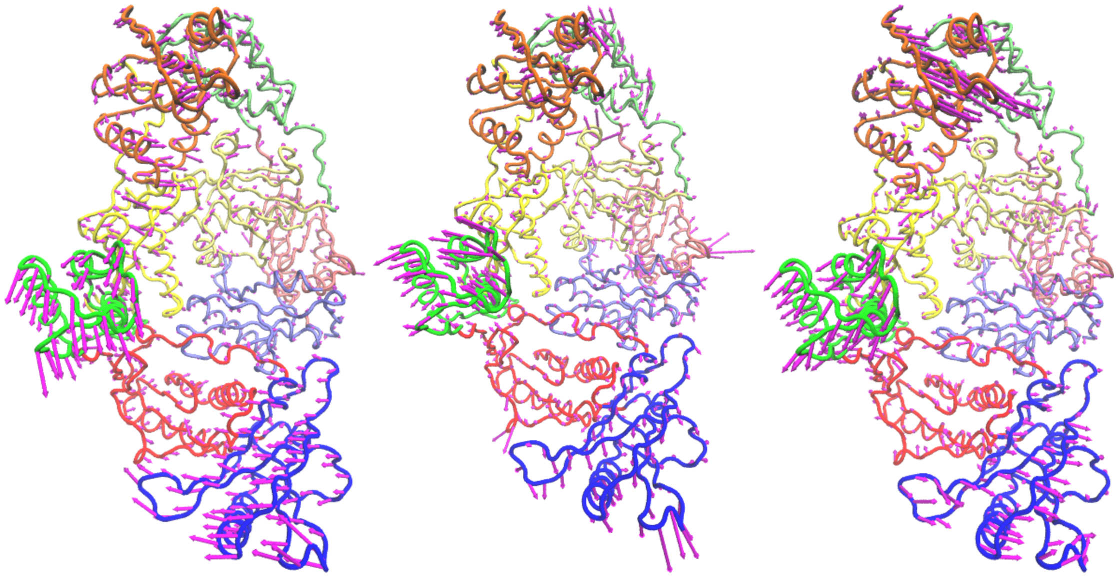

(**a**) (**b**) (**c**)

Figure S6 shows the distribution of the two sidechain dihedral angles (χ_1 and χ_2) of Lys395 in the simulation ensembles of the EFV, NVP bound and apo enzyme. No differentiation can be made between the NNRTI bound and apo systems.

Figure S6. Histogram showing the normalized frequency distribution of the sidechain dihedral angles (**a**) χ_1 and (**b**) χ_2 of Lys395 over the simulations of the apo (black), EFV (red) bound and NVP (blue) bound HIV-1 RT ensemble trajectories. No significant differences are exhibited between the structures.

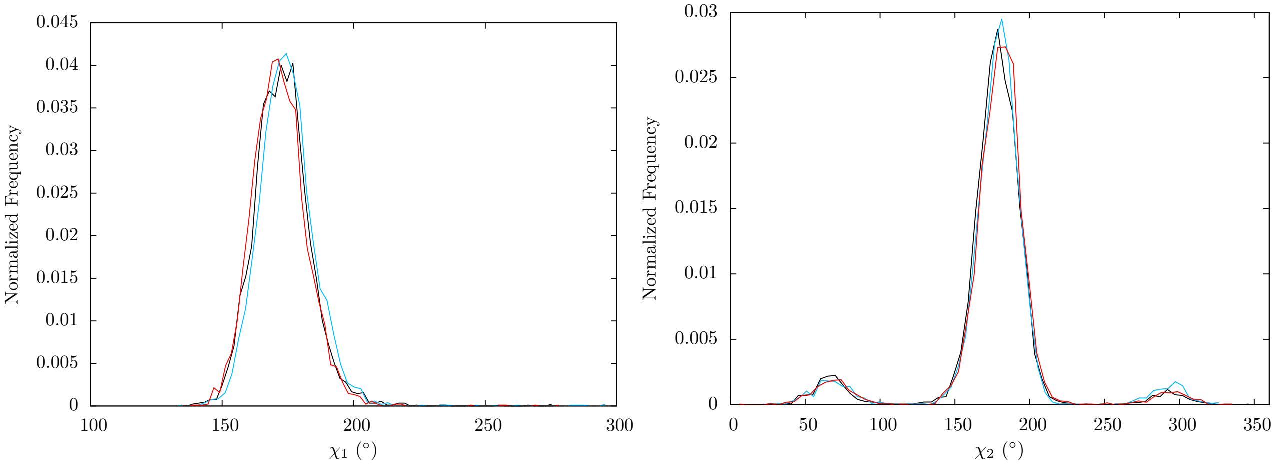

(**a**) (**b**)

References

1. Jacobo-Molina, A.; Ding, J.; Nanni, R.G.; Clark, A.D.; Lu, X.; Tantillo, C.; Williams, R.L.; Kamer, G.; Ferris, A.L.; Clark, P. Crystal structure of human immunodeficiency virus type 1 reverse transcriptase complexed with double-stranded DNA at 3.0 Åresolution shows bent DNA. *Proc. Natl. Acad. Sci. U. S. A.* **1993**, *90*, 6320–6324.

2. Keller, P.A.; Leach, S.P.; Luu, T.T.; Titmuss, S.J.; Griffith, R. Development of computational and graphical tools for analysis of movement and flexibility in large molecules. *J. Mol. Gr. Model.* **2000**, *18*, 235–241, 299.
